# Right Frontoinsular Cortex: A Potential Imaging Biomarker to Evaluate T2DM-Induced Cognitive Impairment

**DOI:** 10.3389/fnagi.2021.674288

**Published:** 2021-05-28

**Authors:** Dongsheng Zhang, Yumeng Lei, Jie Gao, Fei Qi, Xuejiao Yan, Kai Ai, Xia Zhe, Miao Cheng, Man Wang, Yu Su, Min Tang, Xiaoling Zhang

**Affiliations:** ^1^Department of MRI, Shaanxi Provincial People’s Hospital, Xi’an, China; ^2^Department of Graduate, Xi’an Medical University, Xi’an, China; ^3^Department of Clinical Science, Philips Healthcare, Xi’an, China

**Keywords:** type 2 diabetes mellitus, salience network, independent component analysis, voxel-based morphometry, neuroimaging

## Abstract

Cognitive impairment in type 2 diabetes mellitus (T2DM) is associated with functional and structural abnormalities in the intrinsic brain network. The salience network (SN) is a neurocognitive network that maintains normal cognitive function, but it has received little attention in T2DM. We explored SN changes in patients with T2DM with normal cognitive function (DMCN) and in patients with T2DM with mild cognitive impairment (DMCI). Sixty-five T2DM patients and 31 healthy controls (HCs) underwent a neuropsychological assessment, independent component analysis (ICA), and voxel-based morphometry (VBM) analysis. The ICA extracted the SN for VBM to compare SN functional connectivity (FC) and gray matter (GM) volume (GMV) between groups. A correlation analysis examined the relationship between abnormal FC and GMV and clinical/cognitive variables. Compared with HCs, DMCN patients demonstrated increased FC in the left frontoinsular cortex (FIC), right anterior insula, and putamen, while DMCI patients demonstrated decreased right middle/inferior frontal gyrus FC. Compared with DMCN patients, DMCI patients showed decreased right FIC FC. There was no significant difference in SN GMV in DMCN and DMCI patients compared with HCs. FIC GMV was decreased in the DMCI patients compared with DMCN patients. In addition, right FIC FC and SN GMV positively correlated with Montreal Cognitive Assessment and Mini-Mental State Examination (MMSE) scores. These findings indicate that changes in SN FC, and GMV are complex non-linear processes accompanied by increased cognitive dysfunction in patients with T2DM. The right FIC may be a useful imaging biomarker for supplementary assessment of early cognitive dysfunction in patients with T2DM.

## Introduction

Diabetes is becoming increasingly common worldwide and is considered a global chronic illness burden in aging societies (Sinclair et al., 2020). Type 2 diabetes mellitus (T2DM) accounts for 90%–95% of all cases of diabetes (Henning, 2018). T2DM not only leads to multiple chronic complications, such as cardiovascular disease, nephropathy, and retinopathy, but it also increases the risk of dementia, as well as the proportion of patients who convert from mild cognitive impairment to dementia (Koekkoek et al., 2015). Currently, it is believed that the neuropathological basis of cognitive impairment in patients with T2DM is related to increased levels of advanced glycation end-products (AGEs) caused by chronic hyperglycemia. Accumulation of AGEs not only leads to inflammation and oxidative stress but also accelerates amyloid-beta and neuronal tau pathologic processes, which promote neurodegeneration (Munch et al., 1998). T2DM-related cognitive impairment is concealed, mainly affecting executive function, including memory, attention, and visuospatial ability (Palta et al., 2014). Cognitive scales are often used to assess cognitive impairment in clinical practice; however, they are relatively subjective, and it is difficult to identify early changes in cognitive impairment using these tools. Therefore, exploration of biomarkers that can evaluate cognitive decline in patients with T2DM would guide early clinical diagnosis and treatment, as well as prevent or delay cognitive dysfunction and dementia.

Many neuroimaging studies have shown that cognitive impairment is related to structural and functional abnormalities in brain networks (Macpherson et al., 2017; Rosenberg et al., 2019). Among the intrinsic brain networks, the default mode network (DMN), the executive control network (ECN), and the salience network (SN) are considered as the three core neurocognitive networks (Li et al., 2019). The DMN is mainly associated with self-referential mental processes, while the ECN is involved in the maintenance and manipulation of information in working memory, decision making, and goal-directed behavior (Sridharan et al., 2008; Davey et al., 2016). The SN mainly consists of the anterior cingulate cortex and the frontoinsular cortex (FIC), which participate in cognition, emotion, and the internal environment (Seeley et al., 2007). Thus, the SN maintains normal cognitive function and has attracted much attention in recent years (Menon, 2011). One study found that structural and functional impairments in the SN FIC impair one’s ability to identify salient stimuli and reduce perception capacity, which may result in impaired cognitive function (He et al., 2014). The SN is abnormal in patients with Alzheimer’s disease (AD) and frontotemporal dementia. Particularly in the early stages of AD, the SN demonstrates characteristic changes (Zhou et al., 2010).

Previous studies have explored the relationship between cognitive function and intra- and inter-network disconnection, such as in the DMN, the ECN, and the attention network, in patients with T2DM (Cui et al., 2015; Xia et al., 2015; Yang et al., 2016). However, there is no research on the relationship between changes in intra-SN functional connectivity (FC) and cognitive function in patients with T2DM. Although multiple neuroimaging studies (Liu et al., 2017; Wu et al., 2017; Roy et al., 2020) have found that gray matter (GM) volume (GMV) atrophy in the SN core region (insula) is related to cognitive function, the results of research examining functional changes in the SN are not consistent in patients with T2DM. Research using resting-state functional magnetic resonance imaging (fMRI) found that neuronal activity increases in the anterior cingulate cortex of patients with T2DM (Liu et al., 2016), the degree centrality of the right insula and the dorsal anterior cingulate increase, and FC is enhanced (Cui et al., 2016). In addition, patients with T2DM also display increased nodal efficiency in the left insula and right anterior cingulate gyrus (Qin et al., 2019; Xu et al., 2019). These studies speculate that enhancement of neural activity in core regions of the SN in patients with T2DM may be related to compensation of early cognitive impairment. However, Zhou et al. (2014) found that the neural activity of the bilateral insula is decreased in patients with T2DM with mild cognitive impairment (DMCI). Task-based fMRI studies also confirmed that DMCI patients have reduced activation intensity in the right insula and left caudate nucleus under working memory load (Chen et al., 2014). Dai et al. (2017) found that the clustering coefficient and short feature path length of the insula and parahippocampal gyrus are reduced in patients with T2DM with retinal complications, and cognitive impairment is often more severe in patients with T2DM with complications. The heterogeneous results of the above studies may be related to the different cognitive states of subjects. Some studies (Li et al., 2016, 2018) have found that different stages of diabetes-associated cognitive dysfunction exist with different cognitive features. Considering the special role of the SN in cognitive function, it is necessary to explore the relationship between SN changes and cognitive function in patients with T2DM with different cognitive states, which may help us to understand variations in the brain in the context of T2DM-related cognitive impairment.

Independent component analysis (ICA) is a data-driven method that automatically identifies intrinsic connectivity networks in the brain without the need for priori seed regions (Fox and Raichle, 2007). Voxel-based morphometry (VBM) is a fully automated whole-brain measurement technique based on voxel level (Ashburner and Friston, 2000). Therefore, this study aimed to apply ICA and VBM to explore SN changes in patients with T2DM with normal cognitive function (DMCN) and DMCI patients to comprehensively evaluate neuropathological changes in the SN in different cognitive states from both functional and structural viewpoints. We speculate that under different cognitive states, intra-SN FC in patients with T2DM may exhibit different changes, while the GMV of SN-related brain regions in patients with T2DM is consistently atrophied. Abnormally altered FC or GMV in the SN may be related to cognitive function.

## Materials and Methods

### Subjects

One-hundred and five subjects were recruited, including 71 patients diagnosed with T2DM at the Endocrinology Department of Shaanxi Provincial People’s Hospital from May 2018 to July 2019, as well as 34 Healthy controls (HCs), who were examined at the health examination center of our hospital during the same period. All subjects were between 45 and 70 years of age, right-handed, and had at least 6 years of education. The inclusion criteria in the HC group were as follows: (1) no symptoms of T2DM; (2) fasting blood glucose (FBG) concentration of <7.0 mmol/l; (3) glycated hemoglobin (HbA1c) of <6.0%; (4) Mini-Mental State Examination (MMSE) score of ≥27; and (5) Montreal Cognitive Assessment (MoCA) score of ≥26. T2DM was diagnosed according to the criteria proposed by the American Diabetes Association in 2014. Patients with T2DM were on stable therapy (diet, oral medications, and/or insulin). Patients were excluded from the study if they had a history of hypoglycemia (blood glucose concentration of <3.9 mmol/l) or hyperglycemia (blood glucose concentration of >33.3 mmol/l). The exclusion criteria in both groups were as follows: (1) severe claustrophobia or contraindications to MRI; (2) alcoholism, Parkinson’s disease, major depression, brain injury, epilepsy, or other neurological or psychiatric disorders; and (3) any other systemic disease. Then, patients with T2DM were subdivided into the DMCN group and the DMCI group. The inclusion criteria in the DMCI group were as follows: (1) complaints of hypomnesis; (2) MMSE score of >24 and MoCA score of <26; and (3) no other physical or mental disorders that could lead to abnormal cognition.

Every subject arrived at the department for MRI at 6:30–7:00 pm after dinner and controlled their blood glucose according to their doctor’s orders on the day of the scan. MRI was performed after approximately 30 min of structured clinical interview and a series of psychological tests. Only one subject was scanned each day to ensure that each subject had completed the examination with a relatively stable blood glucose. The test procedure and scan time of HCs were the same as those of subjects with T2DM. All subjects were awake during the scan and did not experience discomfort. The study was approved by the Ethics Committee of Shaanxi Provincial People’s Hospital. The study protocol was explained to all subjects, and all subjects provided written informed consent before participation.

### Clinical Data and Neuropsychological Test Information

All subjects underwent the following neuropsychological examinations: MMSE, MoCA, Clock-Drawing Test (CDT), and Trail-Making Test A (TMT-A). The MMSE and MoCA were used to assess general cognitive function. Information processing speed and attention were tested using TMT-A. Executive function and visuospatial skills were evaluated using the CDT. Neuropsychological tests were performed by a psychiatrist with more than 5 years of experience. Clinical data were recorded for all subjects, and clinical data of HCs were collected from the outpatient medical examination center. The medical history and clinical data of subjects were obtained from medical records and questionnaires. Clinical data included blood pressure, height, weight, and body mass index (BMI). Furthermore, HbA1c, FBG concentration, postprandial blood glucose concentration (T2DM patients only), triglyceride (TG) concentration, cholesterol (TC) concentration, and low-density lipoprotein cholesterol (LDL-C) concentration were measured by standard laboratory testing.

### Image Acquisition

MRI data were obtained using a 3.0-T scanner (Ingenia, Philips Healthcare, the Netherlands) and a 16-channel phased-array head coil. All subjects were scanned in a supine head-first position during fMRI, and all subjects were instructed to stay as motionless as possible with their eyes closed, to remain awake, and to think of nothing in particular during scanning. Foam pads and headphones were used to reduce head motion and decrease scanner noise as much as possible during scanning. Additionally, routine T2-weighted imaging and fluid-attenuated inversion recovery (FLAIR) examinations were performed by two radiologists to exclude visible brain lesions. Sagittal three-dimensional T1-weighted imaging was used with the following acquisition parameters: repetition time [TR] = 7.5 ms, echo time [TE] = 3.5 ms, flip angle [FA] = 8°, field of view [FOV] = 250 × 250 mm, matrix = 256 × 256, slice thickness = 1 mm, no gap, and 328 sagittal slices. Resting-state functional blood oxygenation level-dependent images were acquired using a gradient-echo planar sequence with the following parameters: TR = 2,000 ms, TE = 30 ms, slices = 34, thickness = 4 mm, gap = 0 mm, FOV = 230 × 230 mm, matrix = 128 × 128, FA = 90°, and 200 volumes.

### Preprocessing of Structural MRI

The VBM analysis was performed using the Computation Anatomy Toolbox (CAT12^1^), which is based on Statistical Parametric Mapping 12 (SPM12^2^). First, structural MR images were segmented into GM, white matter, and cerebrospinal fluid (CSF), and then segmented GM images were spatially normalized into the Montreal Neurological Institute (MNI) space using DARTEL. After normalization, voxel values within GM images were modulated with the Jacobian determinant of the deformation field. Finally, GM images were smoothed with a full width at a half-maximum (FWHM) kernel of 8 mm. After spatial preprocessing, normalized, modulated, and smoothed maps were used for statistical analysis.

### Preprocessing of Resting-State fMRI Data

All functional images were preprocessed using Data Processing and Analysis for Brain Imaging 3.0^3^, which is based on Statistical Parametric Mapping 12 (SPM12^4^). First, the first 10 volumes were removed to allow subjects to adapt to the magnetic field. Second, slice timing correction was performed to correct for inter-slice time delay within each volume. Third, headmotion >1.5 mm and(or) translation >1.5° of rotation in any direction were excluded. Images were spatially normalized into the MNI space using a standard EPI template provided by SPM12 and resliced into a voxel size of 3 × 3 × 3 mm. Finally, data were spatially smoothed using a 6-mm FWHM Gaussian kernel.

### ICA

The ICA was applied to fMRI data using the group ICA (GICA) of the fMRI toolbox^5^ (MICA version beta 1.2). The toolbox performed the analysis in three stages: (i) a principal component analysis was performed for each subject for data reduction; (ii) the ICA algorithm was applied; and (iii) back-reconstruction was performed for each individual subject. In this study, we performed GICA 100 times, and the number of components (maps and corresponding time courses) estimated for each subject was set to 30. The SN was identified by inspecting aggregate spatial maps and averaging power spectra with four viewers. Individual-level components were obtained from back-reconstruction and converted into *Z*-scores, which reflect the degree to which the time series of a given voxel correlates with the mean time series of its corresponding component. For each SN component, the *Z*-score of each voxel was defined as resting-state intra-network FC. The SN mask identified by ICA is shown in Supplementary Figure 1.

An age-related white matter change scale with a single-blind method was used to quantitatively evaluate lacunar infarcts and white matter hyperintensity (WMH) based on FLAIR recovery images, and subjects with a rating of >2 were excluded. Nine participants were excluded from the final statistical analysis: six subjects (four with T2DM and two HCs) were excluded for excessive motion or poor image quality, and three participants (two with T2DM and one HC) were excluded for a WMH rating of >2. In total, 31 DMCI patients, 34 DMCN patients. and 31 HCs were included in the analyses.

### Statistical Analysis

#### Demographic and Clinical Variable Analysis

Statistical analyses were performed using SPSS 17.0. Analysis of variance (ANOVA) was used to compare demographic data, clinical features, and neuropsychological scores among the three groups. GRF correction and least significant difference (LSD) were used to perform *post hoc* comparisons. The *χ*^2^ test was used to compare proportions, and an independent two-sample *t-test* was used to assess T2DM duration and postprandial blood glucose concentration. *P*-values were considered significant at < 0.05 (for GRF correction, the voxel *P*-value was set to 0.001, and the cluster *P*-value was set to 0.05).

#### FC and GMV Analyses in the SN

Component maps were entered into a random-effect one-sample *t*-test to create a sample-specific component map (*P* < 0.05, GRF-corrected), and the union algorithm was used to combine the three groups’ statistical *Z*-scores, which was used as the mask for subsequent analysis. One-way ANOVA was used to test FC and GMV differences among groups within the SN mask, while GRF correction and LSD were used to perform *post hoc* comparisons.

#### Correlation Analysis

Pearson’s correlation analysis was performed to compare FC and GMV within the SN and neuropsychological test scores and clinical results in patients with T2DM. Mean *Z*-scores for FC and GMV of each brain area that showed significant differences were extracted using Data Processing and Analysis for Brain Imaging 3.0^6^. Pearson’s correlation coefficients between FC, GMV, cognitive performance, and clinical variables were subsequently analyzed using SPSS 17.0. A *P*-value of < 0.05 was considered statistically significant.

## Results

### Clinical and Neuropsychological Data

Demographic, clinical, and neuropsychological data are presented in Table 1. There were no significant differences between groups in age, sex, education level, BMI, blood pressure, TG concentration, TC concentration, LDL-C concentration, and CDT score (*P* > 0.05). As expected, patients with T2DM had higher levels of HbA1c and FBG compared with HCs (all *P* < 0.001). In terms of cognitive performance, DMCI patients had poorer MMSE and MoCA scores (all *P* < 0.001) and higher TMT-A scores compared with DMCN patients and HCs (*P* < 0.05).

**Table 1:** Demographic, clinical, and neuropsychological test results.

	HC (*n* = 31)	DMCN (*n* = 34)	DMCI (*n* = 31)	*F*/*χ*^2^ value	*P*-value
Male/female	18/13	22/12	17/14	0.69	0.71^#^
Age (years)	53.42 ± 4.97	53.97 ± 7.60	56.10 ± 6.12	1.54	0.22
Educational level (years)	14.90 ± 2.57	14.00 ± 2.54	13.90 ± 2.24	1.58	0.21
Diabetes duration (years)	-	9.44 ± 5.35	9.26 ± 5.40	-	0.89
BMI (kg/m^2^)	23.92 ± 3.24	25.10 ± 2.75	24.74 ± 2.90	1.17	0.32
Systolic BP (mmHg)	122.90 ± 9.01	126.91 ± 22.97	127.97 ± 16.99	0.73	0.49
Diastolic BP (mmHg)	81.87 ± 5.89	80.74 ± 11.21	82.16 ± 15.85	0.14	0.87
FBG (mmol/l)	5.38 ± 0.78	9.16 ± 3.04^a^	9.12 ± 3.67^a^	13.96	<0.001*
PBG (mmol/l)	-	14.59 ± 6.46	14.03 ± 5.10	-	0.71
HbA1c (%)	5.53 ± 0.60	8.43 ± 1.75^a^	8.00 ± 2.75^a^	21.36	<0.001*
TG (mmol/l)	1.58 ± 0.92	1.80 ± 1.53	1.84 ± 0.95	0.38	0.68
TC (mmol/l)	4.85 ± 1.01	4.60 ± 0.88	4.74 ± 1.21	0.42	0.66
LDL-C (mmol/l)	2.90 ± 0.95	2.60 ± 0.57	2.67 ± 0.85	1.10	0.34
MMSE	28.84 ± 1.16	29.03 ± 1.06	26.45 ± 0.72^ab^	65.31	<0.001*
MOCA	27.24 ± 1.61	27.25 ± 1.08	22.95 ± 2.03^ab^	89.23	<0.001*
CDT	20.97 ± 6.76	19.50 ± 9.46	22.47 ± 7.30	1.11	0.33
TMT-A	68.33 ± 26.83	72.74 ± 29.72	92.68 ± 29.51^ab^	6.30	0.003*
DR (*n*)	-	3	4	0.28	0.596^#^
DPN (*n*)	-	8	10	0.62	0.432^#^
DN (*n*)	-	6	8	0.64	0.424^#^

Note. Data are presented as mean ± standard deviation or number (%) unless otherwise indicated. BMI, body mass index; FBG, fasting blood glucose; PBG, postprandial blood glucose; HbA1c, glycated hemoglobin; TG, triglyceride; TC, total cholesterol; LDL-C, low-density lipoprotein cholesterol; MMSE, Mini-Mental State Examination; MoCA, Montreal Cognitive Assessment; CDT, Clock-Drawing Test; TMT-A, Trail-Making Test A; DR, diabetic retinopathy; DPN, diabetic peripheral neuropathy; DN, diabetic nephropathy. ^#^*P* for the *χ*^2^ test; *P < 0.05; ^a^*post hoc* paired comparisons show significant differences compared with HCs; ^b^*post hoc* paired comparisons show significant differences compared with DMCN patients.

### FC and GMV Differences Within the SN

**Figure 1:**
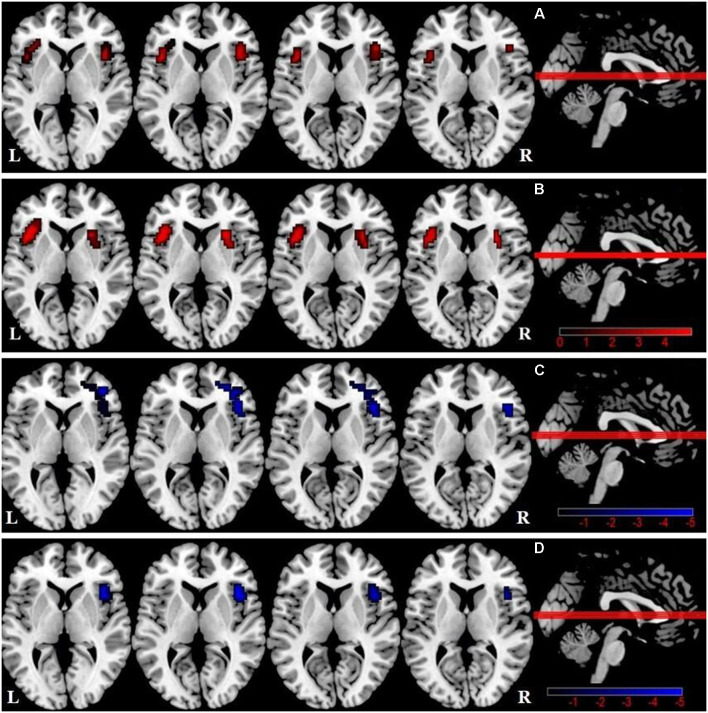
Differences in intra-salience network (SN) functional connectivity (FC; *P* < 0.05, GRF-corrected) among the three groups. **(A)** A one-way analysis of variance (ANOVA) showed brain regions with differences in FC among the three groups. **(B)** Differences in intra-SN FC between DMCN patients and healthy controls (HCs). **(C)** Differences in intra-SN FC between DMCI patients and HCs. **(D)** Differences in intra-SN FC between DMCI patients and DMCN patients.

We observed a significant difference in intra-SN FC in the bilateral FIC using an ANOVA (Table 2, Figure 1A). A between-group analysis demonstrated that the DMCN group displayed increased FC in the left FIC, as well as in the right anterior insula and putamen compared with HCs (Table 2, Figure 1B). The DMCI group showed decreased FC in the right middle/inferior frontal gyrus compared with HCs (Table 2, Figure 1C). Compared with the DMCN group, the DMCI group showed decreased FC in the right FIC (Table 2, Figure 1D).

**Table 2:** Group differences of intra-SN FC among the three groups.

Brain region	BA	Voxels (mm^3^)	Peak MNI coordinates			*t*-value	*P*-value
			X	Y	Z		
ANOVA							
R FIC	13/45	28	42	24	6	12.18	<0.05
L FIC	13/45	29	−42	15	6	13.3	<0.05
HC < DMCN							
L FIC	13/45	89	42	15	6	4.96	<0.05
R insula/putamen		26	27	18	6	4.93	<0.05
HC > DMCI							
R middle/inferior frontal gyrus	10/45	46	42	45	3	−4.45	<0.05
DMCN > DMCI							
R FIC	13	36	42	18	6	−5.01	<0.05

*BA, Brodmann’s area; MNI, Montreal Neurological Institute; FIC, frontoinsular cortex; L, left; R, right*.

**Figure 2:**
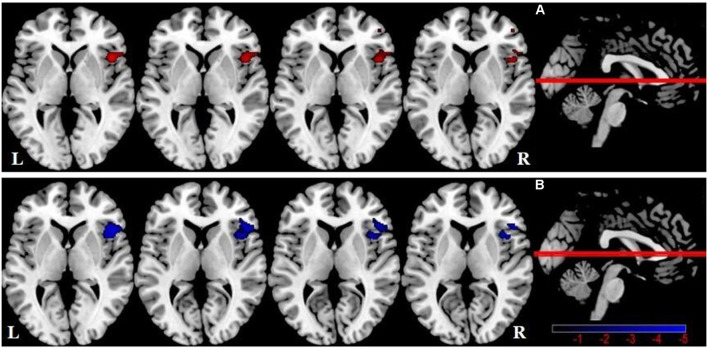
Salience network brain regions with differences in gray matter volume (GMV) among the three groups (*P* < 0.05, GRF-corrected). **(A)** A one-way ANOVA showed SN brain regions with GMV differences between the three groups. **(B)** Differences in GMV within the SN between DMCI patients and DMCN patients.

One-way ANOVA showed significant GMV differences among groups within the SN mask, including the right FIC (Table 3, Figure 2A). Compared with HCs, there was no significant difference in SN GMV between the DMCN and DMCI groups. Compared with the DMCN group, GMV in the right FIC was decreased in the DMCI group (Table 3, Figure 2B). Additionally, the overlapping region where both FC and GMV were different between the DMCN and DMCI patients within SN is shown in Supplementary Figure 2.

**Table 3:** Demographic, clinical, and neuropsychological test results.

Brain region	BA	Voxels (mm^3^)	Peak MNI Coordinates			*t*-value	*P*-value
			X	Y	Z		
ANOVA
R FIC	47/13/45	298	45	18	−1.5	14.36	<0.05
DMCN > DMCI							
R FIC	47/13/45	605	45	18	−1.5	−5.05	<0.05

*BA, Brodmann’s area; MNI, Montreal Neurological Institute; FIC, frontoinsular cortex; L, left; R, right*.

### Correlation Analysis

**Figure 3:**
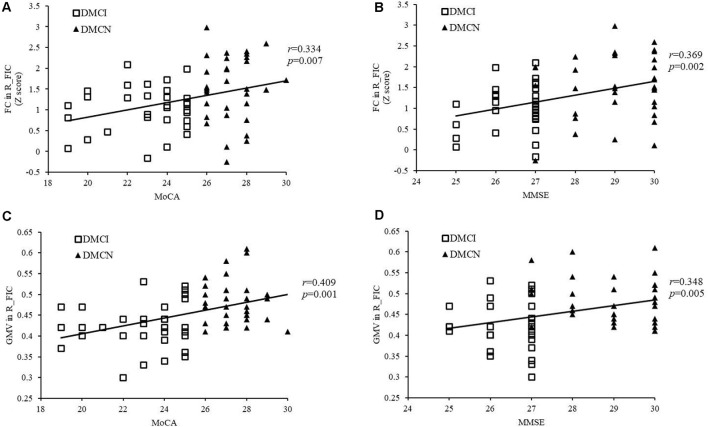
Correlation between the right FIC and cognitive scores. **(A)** Correlation between FC in the right SN FIC and the MoCA score of patients with T2DM (*r* = 0.334, *P* = 0.007). **(B)** Correlation between FC in the right SN FIC and the Mini-Mental State Examination (MMSE) score of patients with T2DM (*r* = 0.369, *P* = 0.002). **(C)** Correlation between GMV in the right SN FIC and the MoCA score of patients with T2DM (*r* = 0.409, *P* = 0.001). **(D)** Correlation between GMV in the right SN FIC and the MMSE score of patients with T2DM (*r* = 0.348, *P* = 0.005).

**Figure 4:**
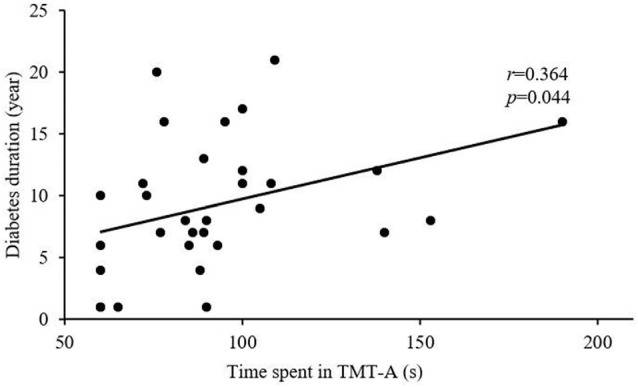
Correlation between TMT-A score and T2DM duration (*r* = 0.364, *P* = 0.044).

After Bonferroni correction for *P*, FC in the right FIC significantly correlated with MoCA and MMSE scores for all T2DM subjects (*r* = 0.334, *P* = 0.007 and *r* = 0.369, *P* = 0.002, respectively; Figures 3A,B), and MoCA and MMSE scores positively correlated with GMV in the right FIC (*r* = 0.409, *P* = 0.001 and *r* = 0.348, *P* = 0.005, respectively; Figures 3C,D). In addition, the T2DM duration in the DMCI group positively correlated with TMT-A scores (*r* = 0.364, *P* = 0.044; Figure 4).

## Discussion

This study reveals altered intra-SN FC and GMV in patients with T2DM under different cognitive states and clarifies the relationship between SN changes and cognitive function. The novel aspect of our research is that DMCI patients demonstrated weakened intra-SN FC and reduced GMV, while DMCN patients exhibited enhanced intra-SN functional coupling. In addition, right FIC FC and GMV in the SN were related to general cognitive function in all patients with T2DM. Our results support the hypothesis that with continuous impairment of cognitive function, the SN, regardless of FC or GMV, may exhibit a complex non-linear pattern of change.

### Intra-SN FC and GMV Changes in DMCN Patients

The SN integrates all kinds of perceptual information from inside and outside the body and identifies the most salient stimulation signals to guide the brain’s activities (Seeley et al., 2007). It is one of the important networks that maintains efficient allocation of brain resources. The FIC and putamen play more integral roles in cognition and executive function (Balleine and O’Doherty, 2010; He et al., 2014). Although DMCN patients did not show obvious cognitive impairment, there were still extensive abnormalities in the brain network (Xiong et al., 2020) and in different cognitive domains (van den Berg et al., 2009; Palta et al., 2014). Van Bussel et al. (2016) found that even in patients with pre-diabetes, there is extensive network reorganization in the brain, which mainly manifests as increased local efficiency, suggesting a functional reorganization of cerebral networks as a compensatory mechanism to cognitive decrement. A previous study (Cai et al., 2020) showed that compared with HCs, subjective cognitive decline showed increased intra-SN FC in the insula and caudate nucleus, hinting that even individuals with normal cognitive function show a compensatory increase in intra-SN activity with self-perceived cognitive decline. In addition, a recent study (Lin et al., 2017) on AD found that increased neuronal activity in the left FIC can counteract abnormal pathological changes caused by early AD and plays a key role in maintaining normal cognitive function. Therefore, we speculate that elevated FC in the intra-SN core node in DMCN patients may serve as a protective mechanism to compensate for existing pathological damage before the clinical manifestation of MCI in patients with T2DM. However, a structural analysis found no significant difference in SN GMV between DMCN patients and HCs, which may indicate that during the period of increased neurological compensation, the gray matter structure does not change significantly, which is consistent with the view that functional changes precede structural damage in cognitive impairment-related diseases.

### Intra-SN FC and GMV Changes in DMCI Patients

The typical pathological basis of cognitive dysfunction in AD is the deposition of β-amyloid and tau proteins (Lemche, 2018). Studies have found that the SN is also one of the brain areas where β-amyloid accumulates (Schultz et al., 2020). T2DM and MCI are both predispositions for AD and have similar neuropathological mechanisms (Bedse et al., 2015; Gibas, 2017). In addition, insulin resistance and high glucose in patients with diabetes also accelerate β-amyloid deposition (Verdile et al., 2015), including in SN core nodes. One study (Jagust and Mormino, 2011) showed that accelerated β-amyloid deposition could lead to premature interruption of compensatory frontal processes, which may be the reason DMCI patients showed reduced FC in the right FIC compared with both HCs and DMCN patients.

This study found that unlike the enhanced functional coupling within the SN of DMCN patients, DMCI patients may have an impairment (decompensation) in the SN, which is consistent with the results of Yang et al. (2016), who found that the right insula is one of the most severely damaged nodes in patients with DMCI. A previous study (He et al., 2014) has shown that the reduction in intra-SN FC in the bilateral FIC of patients with AD is related to cognitive impairment, and intra-SN changes may be a biological marker of AD (Cai et al., 2020). This study observed decreased FC in the right FIC of the SN in DMCN patients, which may indicate that with the decline in cognitive function, an intra-SN disorder in patients with T2DM may show a similar damage pattern to AD.

Another novel finding of this study is that compared with HCs, SN GMV in DMCI patients was no different, but compared with DMCN patients, right FIC GMV in DMCI patients was reduced. T2DM is accompanied by low-grade neuroinflammation (Spranger et al., 2003), which is involved in cognitive impairment associated with T2DM (Marioni et al., 2010) and appears in the early stages (Gispert et al., 2016b). Early neuroinflammation causes extracellular edema and leads to a relatively large GMV (Schwartz et al., 2006; Sykova and Nicholson, 2008), while long–term neuroinflammation eventually leads to neurodegenerative changes, GMV atrophy, and cognitive dysfunction (Daulatzai, 2014). Previous studies have found that the neuroinflammation marker CSF YKL-40 showed an inverted u-shaped association with insular and inferior frontal gyrus GMV in patients with early AD (Gispert et al., 2016a). Therefore, we speculate that DMCN patients may have gray matter edema due to early neuroinflammation, which leads to further aggravation of GMV differences in DMCN patients. The difference in GMV between the DMCI and HC groups was not obvious. This suggests that SN GMV in patients with T2DM may not be a linear process with a continuous decrease in cognitive impairment. In addition, this study found that SN structure and function in DMCI patients have a high degree of overlap, which not only confirms that the right FIC is the key hub for SN disorder in DMCI patients, but also further verifies that structural damage is the basis for dysfunction.

### Correlation Between Intra-SN FC, GMV Changes, and Clinical/Cognitive Variables in Patients With T2DM

The SN has obvious right dominance (Ham et al., 2013; Zhang et al., 2019), the right FIC is the core hub of the SN, which plays an important role in maintaining normal cognition and adjusting the relationship between the CEN and the DMN (Sridharan et al., 2008; He et al., 2014). A meta-analysis (Pan et al., 2017) of MCI found that spontaneous neuronal activity of the bilateral FIC was robustly reduced in patients. It is speculated that the low efficiency of cognitive processes in patients with MCI may be related to an imbalance between networks caused by FIC dysfunction. Zhou et al. (2010) found that decreased right FIC FC is related to the clinical severity of frontotemporal dementia. They believe that the altered characteristics of this inherent network may become non-invasive biomarkers for disease monitoring. Our research found that FIC FC, and GMV in all patients with T2DM are positively correlated with MMSE and MoCA scores. Both the MMSE and MoCA can effectively assess comprehensive cognitive function from different levels, and this correlation may indicate altered FC and GMV in the right FIC, which may, in turn, reflect the degree of cognitive impairment in patients with T2DM. Therefore, we speculate that the FIC may serve as a potential imaging marker to assess cognitive impairment in patients with T2DM.

MCI is a transitional level between the normal brain state and dementia (Albert and Blacker, 2006; Odawara, 2012). It is characterized by decreased memory and attention (Eshkoor et al., 2015). This study shows that DMCI patients have significantly higher TMT-A scores compared with HCs and DMCN patients, which also confirms that DMCI patients have impaired attention. In addition, the correlation analysis found that disease duration positively correlated with TMT-A score in the DMCI group, which may indicate that as the disease duration increases, attention as a function in patients with T2DM gradually decreases. This is consistent with the results of previous studies, which show that as the duration of T2DM increases, cognitive dysfunction worsens (Hu et al., 2019; Liu et al., 2019).

Several limitations of this study should be mentioned. First, the treatment plan for T2DM differed between patients. Different drugs may have had a certain bias on the study results, but this would be difficult to avoid. Second, this experiment has a cross-sectional design and a relatively small sample, therefore, a longitudinal self-controlled study with a large sample would provide strong evidence for our speculation of intra-SN compensation mechanisms. Third, due to the lack of diffusion tensor imaging (DTI) data, we are unable to explore the variation characteristics of SN-related white matter microstructural in patients with T2DM. In future studies, we will collect and analyze DTI data to fully clarify the relationship between SN change patterns and cognitive functions under different cognitive states.

## Conclusion

In conclusion, this study combined ICA and VBM to explore structural and functional changes in the SN in patients with T2DM under different cognitive states. Intra-SN FC and GMV changes differ in DMCN and DMCI patients. Changes in intra-SN FC and GMV are non-linear and complex in patients with T2DM and cognitive impairment. Changes in intra-SN FC may occur through a dynamic process that progresses from compensation to decompensation. More importantly, this study found that the right FIC may be a neuroimaging substrate susceptible to T2DM-related cognitive impairment. This will provide a useful imaging biomarker for the supplementary assessment of cognitive impairment, particularly early recognition of cognitive dysfunction, in patients with T2DM.

## Data Availability Statement

The original contributions presented in the study are included in the article/Supplementary Material, further inquiries can be directed to the corresponding author/s.

## Ethics Statement

The studies involving human participants were reviewed and approved by The ethics committee of Shaanxi Provincial People’s Hospital. The patients/participants provided their written informed consent to participate in this study.

## Author Contributions

DZ drafted the manuscript and designed the experiment. YL performed the statistical analysis. JG contributed to performing the experiment and revised the manuscript. FQ, XY, MW, and YS collected the data. XZ, KA, and MC provided technical support. MT contributed to the manuscript review and critique. XZ made contributions to the design of the experiment and revised the manuscript. All authors contributed to the article and approved the submitted version.

## Conflict of Interest

The authors declare that the research was conducted in the absence of any commercial or financial relationships that could be construed as a potential conflict of interest.

## Footnotes

^1^http://www.neuro.uni-jena.de/cat

^2^http://www.fil.ion.ucl.ac.uk/spm

^3^http://rfmri.org/dpabi

^4^http://www.fil.ion.ucl.ac.uk/spm

^5^http://www.nitrc.org/projects/cogicat/

^6^http://rfmri.org/dpabi

## Abbreviations

AD: Alzheimer’s disease
AGEs: glycation end-products
BMI: body mass index
CDT: Clock-Drawing Test
DMCN: T2DM with normal cognitive function
DMCI: T2DM with mild cognitive impairment
DMN: default-mode network
ECN: executive control network
FBG: fasting blood glucose
FC: functional connectivity
fMRI: functional magnetic resonance imaging
FIC: frontoinsular cortex
GMV: gray matter volume
HbA1c: glycated hemoglobin
HC: healthy controls
ICA: independent component analysis
LDL-C: low-density lipoprotein cholesterol
MMSE: Mini-Mental State Examination
MoCA: Montreal Cognitive Assessment
SN: salience network
T2DM: type 2 diabetes mellitus
TC: cholesterol
TG: triglyceride
TMT-A: Trail Making Test A
VBM: voxel-based morphometry
VSN: visuospatial network.

## References

[B1] Albert M. S. Blacker D. (2006). Mild cognitive impairment and dementia. Annu. Rev. Clin. Psychol. 2, 379–388. 10.1146/annurev.clinpsy.1.102803.144039 17716075

[B2] Ashburner J. Friston K. J. (2000). Voxel-based morphometry—the methods. Neuroimage 11, 805–821. 10.1006/nimg.2000.0582 10860804

[B3] Balleine B. W. O’Doherty J. P. (2010). Human and rodent homologies in action control: corticostriatal determinants of goal-directed and habitual action. Neuropsychopharmacology 35, 48–69. 10.1038/npp.2009.131 19776734 PMC3055420

[B4] Bedse G. Di Domenico F. Serviddio G. Cassano T. (2015). Aberrant insulin signaling in Alzheimer’s disease: current knowledge. Front. Neurosci. 9:204. 10.3389/fnins.2015.00204 26136647 PMC4468388

[B5] Cai C. Huang C. Yang C. Lu H. Hong X. Ren F.. (2020). Altered patterns of functional connectivity and causal connectivity in salience subnetwork of subjective cognitive decline and amnestic mild cognitive impairment. Front. Neurosci. 14:288. 10.3389/fnins.2020.00288 32390791 PMC7189119

[B6] Chen Y. Liu Z. Zhang J. Xu K. Zhang S. Wei D.. (2014). Altered brain activation patterns under different working memory loads in patients with type 2 diabetes. Diabetes Care 37, 3157–3163. 10.2337/dc14-1683 25404661

[B7] Cui Y. Jiao Y. Chen H. J. Ding J. Luo B. Peng C. Y.. (2015). Aberrant functional connectivity of default-mode network in type 2 diabetes patients. Eur. Radiol. 25, 3238–3246. 10.1007/s00330-015-3746-8 25903712 PMC4595523

[B8] Cui Y. Li S. F. Gu H. Hu Y. Z. Liang X. Lu C. Q.. (2016). Disrupted brain connectivity patterns in patients with type 2 diabetes. Am. J. Neuroradiol. 37, 2115–2122. 10.3174/ajnr.A4858 27365332 PMC5201447

[B9] Dai H. Zhang Y. Lai L. Hu S. Wang X. Li Y.. (2017). Brain functional networks: correlation analysis with clinical indexes in patients with diabetic retinopathy. Neuroradiology 59, 1121–1131. 10.1007/s00234-017-1900-5 28831531

[B10] Daulatzai M. A. (2014). Role of stress, depression and aging in cognitive decline and Alzheimer’s disease. Curr. Top. Behav. Neurosci. 18, 265–296. 10.1007/7854_2014_350 25167923

[B11] Davey C. G. Pujol J. Harrison B. J. (2016). Mapping the self in the brain’s default mode network. NeuroImage 132, 390–397. 10.1016/j.neuroimage.2016.02.022 26892855

[B12] Eshkoor S. A. Hamid T. A. Mun C. Y. Ng C. K. (2015). Mild cognitive impairment and its management in older people. Clin. Interv. Aging 10, 687–693. 10.2147/CIA.S73922 25914527 PMC4401355

[B13] Fox M. D. Raichle M. E. (2007). Spontaneous fluctuations in brain activity observed with functional magnetic resonance imaging. Nat. Rev. Neurosci. 8, 700–711. 10.1038/nrn2201 17704812

[B14] Gibas K. J. (2017). The starving brain: Overfed meets undernourished in the pathology of mild cognitive impairment (MCI) and Alzheimer’s disease (AD). Neurochem. Int. 110, 57–68. 10.1016/j.neuint.2017.09.004 28899812

[B15] Gispert J. D. Monte G. C. Falcon C. Tucholka A. Rojas S. Sanchez-Valle R.. (2016a). CSF YKL-40 and pTau181 are related to different cerebral morphometric patterns in early AD. Neurobiol. Aging 38, 47–55. 10.1016/j.neurobiolaging.2015.10.022 26827642

[B16] Gispert J. D. Suarez-Calvet M. Monte G. C. Tucholka A. Falcon C. Rojas S.. (2016b). Cerebrospinal fluid sTREM2 levels are associated with gray matter volume increases and reduced diffusivity in early Alzheimer’s disease. Alzheimers Dement. 12, 1259–1272. 10.1016/j.jalz.2016.06.005 27423963

[B17] Ham T. Leff A. De Boissezon X. Joffe A. Sharp D. J. (2013). Cognitive control and the salience network: an investigation of error processing and effective connectivity. J. Neurosci. 33, 7091–7098. 10.1523/JNEUROSCI.4692-12.2013 23595766 PMC6618896

[B18] He X. Qin W. Liu Y. Zhang X. Duan Y. Song J.. (2014). Abnormal salience network in normal aging and in amnestic mild cognitive impairment and Alzheimer’s disease. Hum. Brain Mapp. 35, 3446–3464. 10.1002/hbm.22414 24222384 PMC6869630

[B19] Henning R. J. (2018). Type-2 diabetes mellitus and cardiovascular disease. Future Cardiol. 14, 491–509. 10.2217/fca-2018-0045 30409037

[B20] Hu B. Yan L. F. Sun Q. Yu Y. Zhang J. Dai Y. J.. (2019). Disturbed neurovascular coupling in type 2 diabetes mellitus patients: Evidence from a comprehensive fMRI analysis. Neuroimage Clin. 22:101802. 10.1016/j.nicl.2019.101802 30991623 PMC6447740

[B21] Jagust W. J. Mormino E. C. (2011). Lifespan brain activity, beta-amyloid and Alzheimer’s disease. Trends Cogn. Sci. 15, 520–526. 10.1016/j.tics.2011.09.004 21983147 PMC3206968

[B22] Koekkoek P. S. Kappelle L. J. van den Berg E. Rutten G. E. Biessels G. J. (2015). Cognitive function in patients with diabetes mellitus: guidance for daily care. Lancet Neurol. 14, 329–340. 10.1016/S1474-4422(14)70249-2 25728442

[B23] Lemche E. (2018). Early life stress and epigenetics in late-onset Alzheimer’s dementia: a systematic review. Curr. Genomics 19, 522–602. 10.2174/1389202919666171229145156 30386171 PMC6194433

[B24] Li C. Li C. Yang Q. Wang B. Yin X. Zuo Z.. (2018). Cortical thickness contributes to cognitive heterogeneity in patients with type 2 diabetes mellitus. Medicine 97:e10858. 10.1097/MD.0000000000010858 29794784 PMC6392513

[B25] Li C. Li Y. Zheng L. Zhu X. Shao B. Fan G.. (2019). Abnormal brain network connectivity in a triple-network model of Alzheimer’s disease. J. Alzheimers Dis. 69, 237–252. 10.3233/JAD-181097 30958354

[B26] Li W. Risacher S. L. Huang E. Saykin A. J. (2016). Type 2 diabetes mellitus is associated with brain atrophy and hypometabolism in the ADNI cohort. Neurology 87, 595–600. 10.1212/WNL.0000000000002950 27385744 PMC4977372

[B27] Lin F. Ren P. Lo R. Y. Chapman B. P. Jacobs A. Baran T. M.. (2017). Insula and inferior frontal gyrus’ activities protect memory performance against Alzheimer’s disease pathology in old age. J. Alzheimers Dis. 55, 669–678. 10.3233/JAD-160715 27716674 PMC5531269

[B28] Liu D. Duan S. Zhang J. Zhou C. Liang M. Yin X.. (2016). Aberrant brain regional homogeneity and functional connectivity in middle-aged T2DM patients: a resting-state functional MRI study. Front. Hum. Neurosci. 10:490. 10.3389/fnhum.2016.00490 27729856 PMC5037166

[B29] Liu H. Liu J. Peng L. Feng Z. Cao L. Liu H.. (2019). Changes in default mode network connectivity in different glucose metabolism status and diabetes duration. NeuroImage Clin. 21:101629. 10.1016/j.nicl.2018.101629 30573410 PMC6411780

[B30] Liu J. Liu T. Wang W. Ma L. Ma X. Shi S.. (2017). Reduced gray matter volume in patients with type 2 diabetes mellitus. Front. Aging Neurosci. 9:161. 10.3389/fnagi.2017.00161 28588480 PMC5439076

[B31] Macpherson H. Formica M. Harris E. Daly R. M. (2017). Brain functional alterations in Type 2 diabetes—A systematic review of fMRI studies. Front. Neuroendocrinol. 47, 34–46. 10.1016/j.yfrne.2017.07.001 28687473

[B32] Marioni R. E. Strachan M. W. Reynolds R. M. Lowe G. D. Mitchell R. J. Fowkes F. G.. (2010). Association between raised inflammatory markers and cognitive decline in elderly people with type 2 diabetes: the edinburgh type 2 diabetes study. Diabetes 59, 710–713. 10.2337/db09-1163 19959761 PMC2828661

[B33] Menon V. (2011). Large-scale brain networks and psychopathology: a unifying triple network model. Trends. Cogn. Sci. 15, 483–506. 10.1016/j.tics.2011.08.003 21908230

[B34] Munch G. Schinzel R. Loske C. Wong A. Durany N. Li J. J.. (1998). Alzheimer’s disease–synergistic effects of glucose deficit, oxidative stress and advanced glycation endproducts. J. Neural Transm. (Vienna) 105, 439–461. 10.1007/s007020050069 9720973

[B35] Odawara T. (2012). Cautious notification and continual monitoring of patients with mild cognitive impairment. Psychogeriatrics 12, 131–132. 10.1111/j.1479-8301.2012.00417.x 22712649

[B36] Palta P. Schneider A. L. Biessels G. J. Touradji P. Hill-Briggs F. (2014). Magnitude of cognitive dysfunction in adults with type 2 diabetes: a meta-analysis of six cognitive domains and the most frequently reported neuropsychological tests within domains. J. Int. Neuropsychol. Soc. 20, 278–291. 10.1017/S1355617713001483 24555960 PMC4132660

[B37] Pan P. Zhu L. Yu T. Shi H. Zhang B. Qin R.. (2017). Aberrant spontaneous low-frequency brain activity in amnestic mild cognitive impairment: a meta-analysis of resting-state fMRI studies. Ageing Res. Rev. 35, 12–21. 10.1016/j.arr.2016.12.001 28017880

[B38] Qin C. Liang Y. Tan X. Leng X. Lin H. Zeng H.. (2019). Altered whole-brain functional topological organization and cognitive function in type 2 diabetes mellitus patients. Front. Neurol. 10:599. 10.3389/fneur.2019.00599 31275222 PMC6593281

[B39] Rosenberg J. Lechea N. Pentang G. N. Shah N. J. (2019). What magnetic resonance imaging reveals—A systematic review of the relationship between type II diabetes and associated brain distortions of structure and cognitive functioning. Front. Neuroendocrinol. 52, 79–112. 10.1016/j.yfrne.2018.10.001 30392901

[B40] Roy B. Ehlert L. Mullur R. Freeby M. J. Woo M. A. Kumar R.. (2020). Regional brain gray matter changes in patients with type 2 diabetes mellitus. Sci. Rep. 10:9925. 10.1038/s41598-020-67022-5 32555374 PMC7303156

[B41] Schultz A. P. Buckley R. F. Hampton O. L. Scott M. R. Properzi M. J. Pena-Gomez C.. (2020). Longitudinal degradation of the default/salience network axis in symptomatic individuals with elevated amyloid burden. Neuroimage Clin. 26:102052. 10.1016/j.nicl.2019.102052 31711955 PMC7229343

[B42] Schwartz M. Butovsky O. Bruck W. Hanisch U. K. (2006). Microglial phenotype: is the commitment reversible. Trends. Neurosci. 29, 68–74. 10.1016/j.tins.2005.12.005 16406093

[B43] Seeley W. W. Menon V. Schatzberg A. F. Keller J. Glover G. H. Kenna H.. (2007). Dissociable intrinsic connectivity networks for salience processing and executive control. J. Neurosci. 27, 2349–2356. 10.1523/JNEUROSCI.5587-06.2007 17329432 PMC2680293

[B44] Sinclair A. Saeedi P. Kaundal A. Karuranga S. Malanda B. Williams R. (2020). Diabetes and global ageing among 6599-year-old adults: findings from the international diabetes federation diabetes atlas, 9(th) edition. Diabetes Res. Clin. Pract. 162:108078. 10.1016/j.diabres.2020.108078 32068097

[B45] Spranger J. Kroke A. Mohlig M. Hoffmann K. Bergmann M. M. Ristow M.. (2003). Inflammatory cytokines and the risk to develop type 2 diabetes: results of the prospective population-based european prospective investigation into cancer and nutrition (EPIC)-potsdam study. Diabetes 52, 812–817. 10.2337/diabetes.52.3.812 12606524

[B46] Sridharan D. Levitin D. J. Menon V. (2008). A critical role for the right fronto-insular cortex in switching between central-executive and default-mode networks. Proc. Natl. Acad. Sci. U S A 105, 12569–12574. 10.1073/pnas.0800005105 18723676 PMC2527952

[B47] Sykova E. Nicholson C. (2008). Diffusion in brain extracellular space. Physiol. Rev. 88, 1277–1340. 10.1152/physrev.00027.2007 18923183 PMC2785730

[B48] Van Bussel F. C. Backes W. H. van Veenendaal T. M. Hofman P. A. van Boxtel M. P. Schram M. T.. (2016). Functional brain networks are altered in type 2 diabetes and prediabetes: signs for compensation of cognitive decrements? The maastricht study. Diabetes 65, 2404–2413. 10.2337/db16-0128 27217484

[B49] van den Berg E. Kloppenborg R. P. Kessels R. P. Kappelle L. J. Biessels G. J. (2009). Type 2 diabetes mellitus, hypertension, dyslipidemia and obesity: A systematic comparison of their impact on cognition. Biochim. Biophys. Acta 1792, 470–481. 10.1016/j.bbadis.2008.09.004 18848880

[B50] Verdile G. Fuller S. J. Martins R. N. (2015). The role of type 2 diabetes in neurodegeneration. Neurobiol. Dis. 84, 22–38. 10.1016/j.nbd.2015.04.008 25926349

[B51] Wu G. Lin L. Zhang Q. Wu J. (2017). Brain gray matter changes in type 2 diabetes mellitus: A meta-analysis of whole-brain voxel-based morphometry study. J. Diabetes Complications 31, 1698–1703. 10.1016/j.jdiacomp.2017.09.001 29033311

[B52] Xia W. Wang S. Rao H. Spaeth A. M. Wang P. Yang Y.. (2015). Disrupted resting-state attentional networks in T2DM patients. Sci. Rep. 5:11148. 10.1038/srep11148 26053355 PMC4459168

[B53] Xiong Y. Chen X. Zhao X. Fan Y. Zhang Q. Zhu W. (2020). Altered regional homogeneity and functional brain networks in type 2 diabetes with and without mild cognitive impairment. Sci. Rep. 10:21254. 10.1038/s41598-020-76495-3 33277510 PMC7718881

[B54] Xu J. Chen F. Liu T. Wang T. Zhang J. Yuan H.. (2019). Brain functional networks in type 2 diabetes mellitus patients: a resting-state functional MRI study. Front. Neurosci. 13:239. 10.3389/fnins.2019.00239 30941007 PMC6433793

[B55] Yang S.Q. Xu Z.P. Xiong Y. Zhan Y.F. Guo L.Y. Zhang S.. (2016). Altered intranetwork and internetwork functional connectivity in type 2 diabetes mellitus with and without cognitive impairment. Sci. Rep. 6:32980. 10.1038/srep32980 27622870 PMC5020685

[B56] Zhang Y. Suo X. Ding H. Liang M. Yu C. Qin W. (2019). Structural connectivity profile supports laterality of the salience network. Hum. Brain Mapp. 40, 5242–5255. 10.1002/hbm.24769 31436006 PMC6864895

[B57] Zhou J. Greicius M. D. Gennatas E. D. Growdon M. E. Jang J. Y. Rabinovici G. D.. (2010). Divergent network connectivity changes in behavioural variant frontotemporal dementia and Alzheimer’s disease. Brain 133, 1352–1367. 10.1093/brain/awq075 20410145 PMC2912696

[B58] Zhou X. Zhang J. Chen Y. Ma T. Wang Y. Wang J.. (2014). Aggravated cognitive and brain functional impairment in mild cognitive impairment patients with type 2 diabetes: a resting-state functional MRI study. J. Alzheimers Dis. 41, 925–935. 10.3233/JAD-132354 24705547

