# Supplementary figures and images for: Right Frontoinsular Cortex: A Potential Imaging Biomarker to Evaluate T2DM-Induced Cognitive Impairment
Source: Front Aging Neurosci. 2021 May 28;13:674288. doi: 10.3389/fnagi.2021.674288 (PMC8193040; doi:10.3389/fnagi.2021.674288)

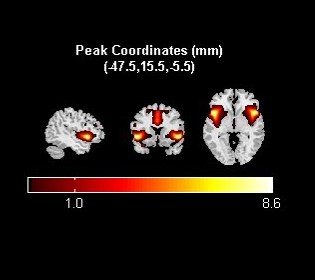

Supplement: Supplementary file 1 [file Image_1.TIFF]

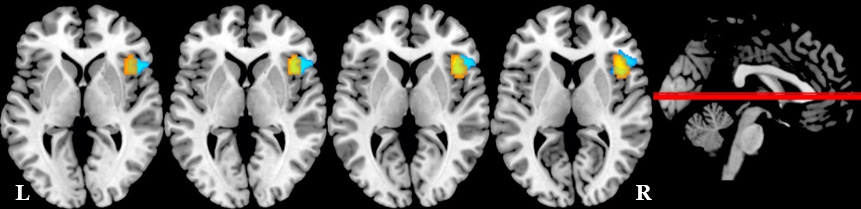

Supplement: Supplementary file 2 [file Image_2.TIFF]
